# Supplementary material for: In vivo single-molecule analysis reveals COOLAIR RNA structural diversity
Source: Nature. 2022 Aug 17;609(7926):394–9. doi: 10.1038/s41586-022-05135-9 (PMC9452300; doi:10.1038/s41586-022-05135-9)

nature portfolio

https://doi.org/10.1038/s41586-022-05135-9

Supplementary information

In vivo single-molecule analysis reveals *COOLAIR* RNA structural diversity

In the format provided by the authors and unedited

Supplementary Discussion

In vivo single-molecule analysis reveals *COOLAIR* RNA structural diversity

Minglei Yang[1,2], Pan Zhu[1,2], Jitender Cheema[1], Rebecca Bloomer[1], Pawel Mikulski[1], Qi Liu[1], Yueying Zhang[1], Caroline Dean[1]* & Yiliang Ding[1]*

[1] John Innes Centre, Norwich Research Park, Norwich, NR4 7UH, UK.

[2] These authors contributed equally: Minglei Yang and Pan Zhu

*Correspondence to: Caroline Dean (caroline.dean@jic.ac.uk) and Yiliang Ding (yiliang.ding@jic.ac.uk)

Solving *in vivo* RNA structure conformation at the single molecule level.

The structure is an intrinsic property of an RNA molecule that serves to provide important functional information beyond its nucleotide sequence. Since 2010, a variety of high-throughput (mostly illumina-based short-read sequencing) RNA structure profiling methods have transformed the scope of RNA structure studies, enabling genome-wide RNA structure analyses[1]. However, there are still three main challenges to decipher the RNA structure *in vivo*.

The isoform heterogeneity is a major challenge to accurately assign RNA structural information to individual gene-linked isoforms. 90% of human genes[2] and 60% *Arabidopsis* genes[2] produce alternatively spliced transcripts. The RNA structural information within the shared regions between isoforms cannot be distinguished by short read sequencing platforms (e.g., Illumina). Our smStructure-seq addresses this challenge by using the PacBio sequencing method. The sequencing principle of the PacBio platform allows the accurate assignment of different transcript isoforms[3–6], since there is no assembly step.

The second challenge is to determine the RNA structural information for single molecules. RNA structures adopt multiple conformations. The single-molecule structural information can not only discriminate RNAs with very similar sequence (e.g, isoform or RNA sub-genome in viruses), but facilitate the identification of RNA structural diversity (the third challenge of RNA structure analysis). Recently, a Nanopore-based method, PORE-cupine[7] was developed to address these challenges. The long-read Nanopore sequencing captures structures along the whole length of each isoform[7]. However, the macromolecules in the Nanopore channel can be occupied by multiple bases at one time, increasing uncertainty in signal assignment of the nucleotides[7,8]. Besides, Nanopore has an averaged error rate of 14% for both direct RNA and cDNA sequencing[9], which cannot achieve the single-molecule accuracy. In contrast, PacBio platform used by smStructure-seq can achieve 99.9% accuracy at the nucleotide level[2], facilitating the accurate derivation of RNA structure for each single RNA molecule. The accurate single-molecule read is the foundation to decipher the conformation diversity at the single-molecule level.

The RNA structure can dynamically change *in vivo* by adopting different conformations. Directly dissecting the diversity of different RNA structural conformations remains challenging. Two new computational approaches, DREEM[10] and DRACO[11] were developed trying to solve this problem. DREEM[10] used an expectation–maximization regime to detect the RNA structure conformation while DRACO[11] used an alternative method based on a new clustering regime. These two computational methods were developed to estimate structural conformations based on the Illumina-based platform. Due to the limitation of short read sequencing, the direct dissection of RNA structural conformations has so far only been achieved for short RNA fragments (200-300nt)[10], although in theory these methods could be

improved for long transcripts. These two computational approaches deduce the RNA structure conformation by clustering the chemical reactivity profiles. The chemical reactivity-based clustering methods tend to generate two mutation profiles with one extreme high chemical modification (more single-stranded RNA structure) and one extreme low chemical modification (more double-stranded RNA structure). These clusters directly reflect the similarity of chemical modification efficiencies rather than directly represent the similarity of RNA structure *per se*.

smStructure-seq can solve these challenges by taking advantage of highly-accurate single-molecule sequencing, together with our new analysis method that directly clusters the *in vivo* RNA structures derived from the mutation profile of each single RNA molecule. This method named **D**etermin**a**tion of the **V**ar**i**atio**n** of RNA structure **c**onformat**i**on (DaVinci), incorporates the individual mutation profiles and derives the most-likely RNA structure conformation via a stochastic context-free grammar (SCFG) algorithm independent of thermodynamic parameters. Then the whole conformation space is identified and visualized via dimensionality reduction analysis, e.g., PCA or MDS (Extended Data Fig. 2a and Methods). Thus, using the DaVinci method we can accurately deduce structural conformation of each single RNA molecule.

To demonstrate the power of DaVinci, we performed our method on the HIV-1 Rev response element (RRE) that has been reported to be able to adopt alternative conformations promoting different rates of virus replication[12]. DREEM used chemical reactivity-based clustering methods and identified two extreme conformations (conformation 1 and conformation 2 in Extended Data Fig. 2d)[10]. However, DaVinci could identify at least three conformations (conformation 1, 2 and 3 in Extended Data Fig. 2b, d) including an extra cryptic conformation[13] i.e., conformation 3 that cannot be identified by chemical reactivity-based clustering methods,

e.g., DREEM[10]. Conformation 3 was first reported in mutant RRE6[13] and has the ability to confer RevM10 resistance[13]. However, this RNA structure is not exclusive to mutant RRE61 but present in wild-type RRE as well. We generate *in silico* RNA structure ensembles on wild-type RRE and mutation RRE61 by Boltzmann sampling (10,000 times) using RNAfold[14]. Three structural clusters (Extended Data Fig. 2b) were found with conformation 3 being the least abundant (1%) in RRE. With the mutant RRE61[13], conformation 3 increased to 95.6% (Extended Data Fig. 2c). Thus, the wild-type RRE sequence has the potential to fold into the rare conformation 3 with the mutation converting it to the dominant conformation.

To experimentally confirm the conformational change caused by the mutations in RRE61, we folded the RRE61 RNA *in vitro* and probed the structure[10]. We then performed our DaVinci analysis and found that conformation 3 increased to 81% (Extended Data Fig. 2e) from 2% in RRE[10] (Extended Data Fig. 2d). DaVinci directly measures the percentage of clusters by counting each single RNA structure derived from the probing data and this contrasts with *in silico* RNA structure ensemble analysis, where Boltzmann sampling measures the percentage using a function of free energy. Thus, DaVinci analysis can estimate accurate proportions and distributions of each conformation cluster. Our analysis also confirms that mutations or single nucleotide polymorphisms (SNP) can alter the RNA structural ensemble and change the proportion of different conformations[15–17].

A second example comes from the analysis of TPP riboswitch, a typical RNA molecule which can fold into alternative structures depending on the presence of the TPP ligand[18–20]. We performed the RNA structure probing experiments on *in vitro* folded TPP riboswitch RNAs (TenA gene in *B. subtilis*) in the absence or presence of TPP ligand. After the treatment of the SHAPE chemicals (NAI), we merged the NAI-modified RNA samples (TPP-treated and non

TPP-treated RNAs) with a ratio of 20:80 (vol/vol) or 50:50 (vol/vol) and conducted the library constructions, respectively. We then performed our DaVinci analysis on the obtained sequencing data and found that DaVinci closely reflects the different ratio of the two alternative conformations (Conformation 1 is related to the TPP-treated conformation and Conformation 2 is related to the non TPP-treated one) with the ratios of 29:71 or 40:60 (Extended Data Fig. 2f-h). Overall, DaVinci accurately detects RNA structural conformations. The slight difference between the expected ratios and the DaVinci-derived ratios is likely to reflect the equilibrium of the conformations during the RNA structure probing in the solutions.

To further test DaVinci, we exploited a published dataset on RNA structure probing of Escherichia coli cspA 5' untranslated region (UTR). This UTR functions as an RNA thermometer since it can switch states between translationally repressed conformations (conformation 3 and 4) at 37 °C and translationally competent conformations (conformation 1 and 2) at 10 °C[21,22]. DaVinci results showed that the translationally competent conformations (conformation 1 and 2) increased from 23% to 67% upon transfer from 37 °C to 10 °C. These two conformations have been previously detected after the cold treatments[22]. DaVinci also identified an extra conformation 3, which is very similar to the major conformation 4[22] at 37 °C. Compared with the conformation 4, conformation 3 loses a short stem loop, further indicating that DaVinci is sensitive to detect less abundant RNA structural conformations.

These results showed that DaVinci can identify the dynamic nature of *in vivo* RNA structure conformations, facilitating the investigation of the RNA structural conformation functionality *in vivo*.

Therefore, our smStructure-seq allied with DaVinci analysis pipeline can address the challenges of both heterogeneities of isoforms and structural conformations simultaneously and thus is capable of generating single-molecule RNA structure conformations for each RNA transcript (e.g., isoform).

References

1. Zhang, H. & Ding, Y. Novel insights into the pervasive role of RNA structure in post-transcriptional regulation of gene expression in plants. *Biochem. Soc. Trans.* **49**, 1829–1839 (2021).
2. Pan, Q., Shai, O., Lee, L. J., Frey, B. J. & Blencowe, B. J. Deep surveying of alternative splicing complexity in the human transcriptome by high-throughput sequencing. *Nat. Genet.* **40**, 1413–1415 (2008).
3. Zhao, L. *et al.* Analysis of transcriptome and epitranscriptome in plants using PacBio Iso-Seq and Nanopore-based direct RNA sequencing. *Front. Genet.* **10**, 1–14 (2019).
4. An, D., Cao, H., Li, C., Humbeck, K. & Wang, W. Isoform sequencing and state-of-art applications for unravelling complexity of plant transcriptomes. *Genes (Basel).* **9**, 43 (2018).
5. Mays, A. D. *et al.* Single-molecule real-time (SMRT) full-length RNA-sequencing reveals novel and distinct mRNA isoforms in human bone marrow cell subpopulations. *Genes (Basel).* **10**, 253 (2019).
6. Wenger, A. M. *et al.* Accurate circular consensus long-read sequencing improves variant detection and assembly of a human genome. *Nat. Biotechnol.* **37**, 1155–1162 (2019).
7. Aw, J. G. A. *et al.* Determination of isoform-specific RNA structure with nanopore long reads. *Nat. Biotechnol.* **39**, 336–346 (2021).

8. Branton, D. *et al.* The potential and challenges of nanopore sequencing. *Nature Biotechnology* **26**, 1146–1153 (2008).

9. Workman, R. E. *et al.* Nanopore native RNA sequencing of a human poly(A) transcriptome. *Nat. Methods* **16**, 1297–1305 (2019).

10. Tomezsko, P. J. *et al.* Determination of RNA structural diversity and its role in HIV-1 RNA splicing. *Nature* **582**, 438–442 (2020).

11. Morandi, E. *et al.* Genome-scale deconvolution of RNA structure ensembles. *Nat. Methods* **18**, 249–252 (2021).

12. Sherpa, C., Rausch, J. W., Le Grice, S. F. J., Hammarskjold, M. L. & Rekosh, D. The HIV-1 Rev response element (RRE) adopts alternative conformations that promote different rates of virus replication. *Nucleic Acids Res.* **43**, 4676–4686 (2015).

13. Legiewicz, M. *et al.* Resistance to RevM10 inhibition reflects a conformational switch in the HIV-1 Rev response element. *Proc. Natl. Acad. Sci. U. S. A.* **105**, 14365–14370 (2008).

14. Hofacker, I. *et al.* Fast folding and comparison of RNA secondary structures. *Monatsh Chem (Chem Mon.* **125**, 167–188 (1994).

15. Halvorsen, M., Martin, J. S., Broadaway, S. & Laederach, A. Disease-associated mutations that alter the RNA structural ensemble. *PLoS Genet.* **6**, e1001074 (2010).

16. Martin, J. S. *et al.* Structural effects of linkage disequilibrium on the transcriptome. *RNA* **18**, 77–87 (2012).

17. Linnstaedt, S. D. *et al.* A functional riboSNitch in the 3′ untranslated region of FKBP5 alters microRNA-320a binding efficiency and mediates vulnerability to chronic post-traumatic pain. *J. Neurosci.* **38**, 8407–8420 (2018).

18. Edwards, T. E. & Ferré-D'Amaré, A. R. Crystal structures of the thi-box riboswitch bound to thiamine pyrophosphate analogs reveal adaptive RNA-small molecule

recognition. *Structure* **14**, 1459–1468 (2006).

19. Quarta, G., Kim, N., Izzo, J. A. & Schlick, T. Analysis of riboswitch structure and function by an energy landscape framework. *J. Mol. Biol.* **393**, 993–1003 (2009).

20. Manzourolajdad, A. & Arnold, J. Secondary structural entropy in RNA switch (Riboswitch) identification. *BMC Bioinformatics* **16**, (2015).

21. Giuliodori, A. M. *et al.* The cspA mRNA is a thermosensor that modulates translation of the cold-shock protein cspA. *Mol. Cell* **37**, 21–33 (2010).

22. Zhang, Y. *et al.* A stress response that monitors and regulates mRNA structure is central to cold shock adaptation. *Mol. Cell* **70**, 274-286.e7 (2018).

Extended Data Fig.8a

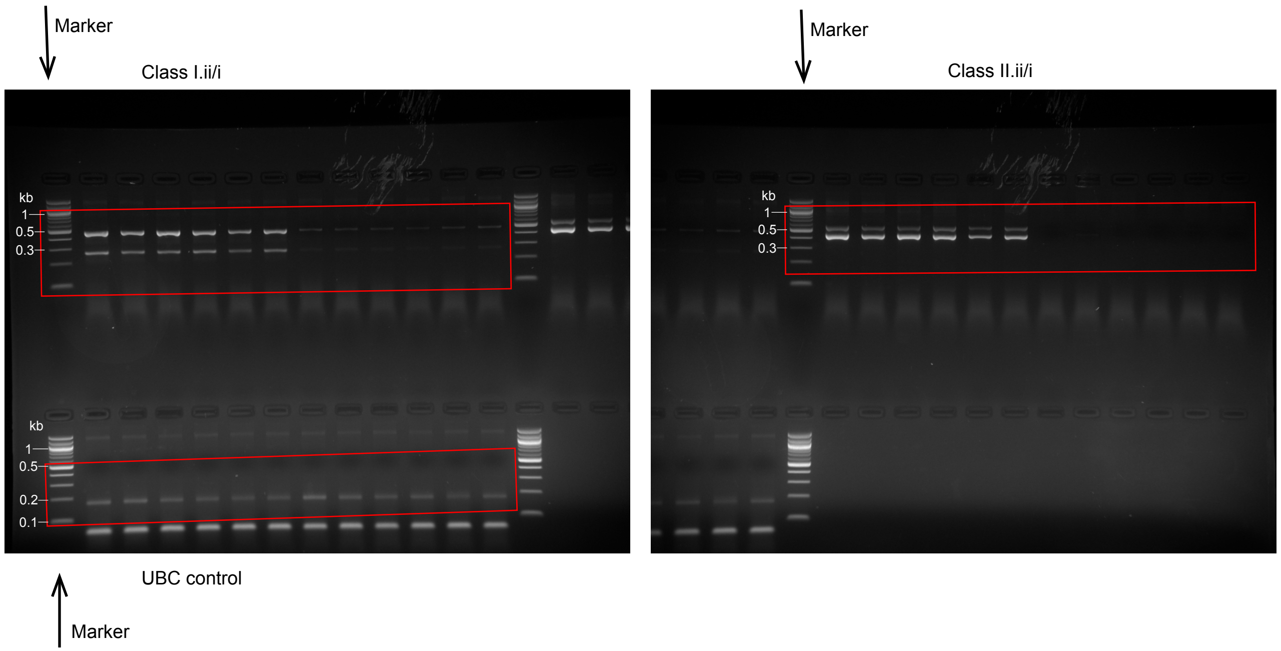

Extended Data Fig. 9

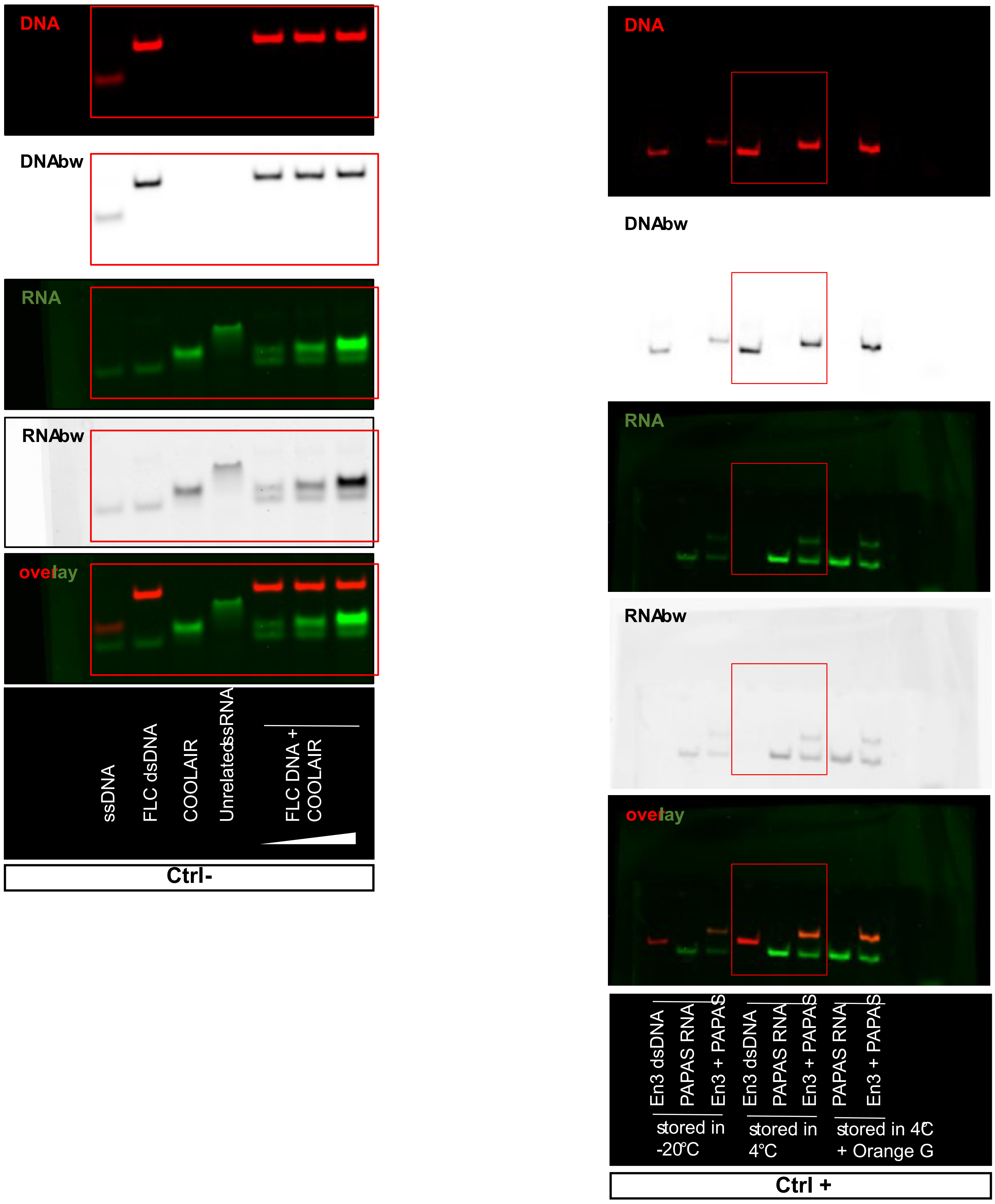

Extended Data Fig. 9

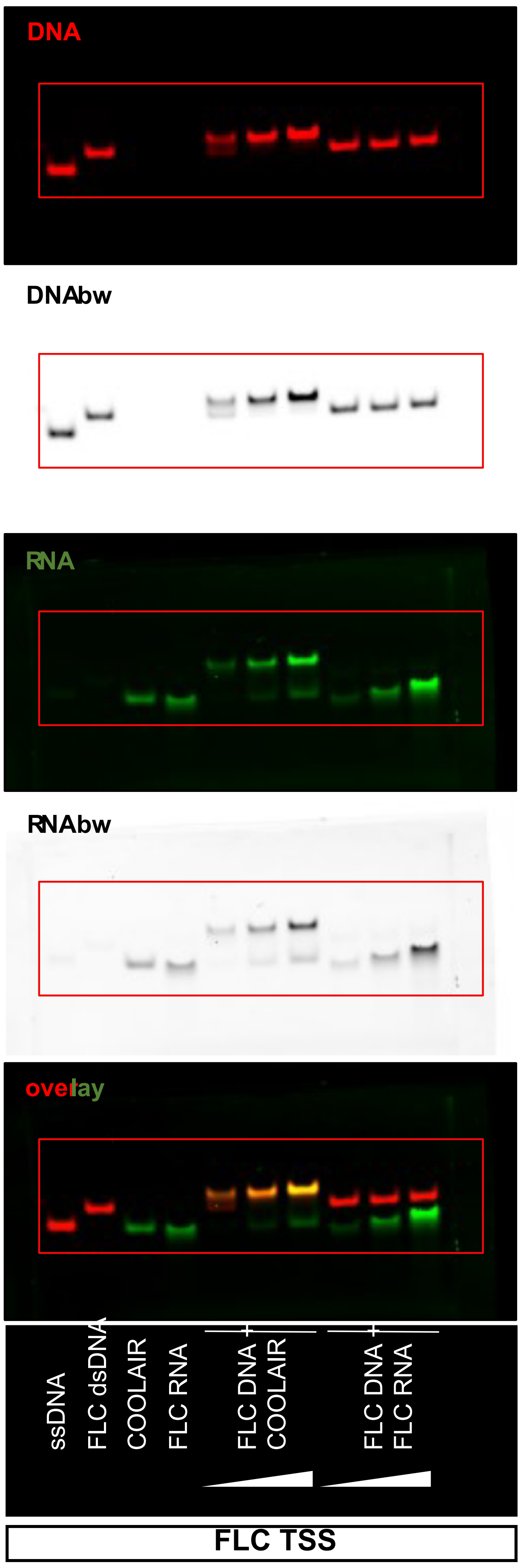

Supplement: Supplementary file 1 — The Supplementary Discussion includes details about the advantage of smStructure-seq and the validation of DaVinci methods. Supplementary Figure 1 contains original source images for RT–qPCR of the spliced class I and class II COOLAIR isoforms as well as the EMSA for triplex formation. [file 41586_2022_5135_MOESM1_ESM.pdf]
