# Supplementary material for: In vivo single-molecule analysis reveals COOLAIR RNA structural diversity
Source: Nature. 2022 Aug 17;609(7926):394–9. doi: 10.1038/s41586-022-05135-9 (PMC9452300; doi:10.1038/s41586-022-05135-9)
Supplement: Supplementary file 2 — Reporting Summary [file 41586_2022_5135_MOESM2_ESM.pdf]

nature research

Corresponding author(s): Caroline Dean & Yiliang Ding

Last updated by author(s): Jun 13, 2022

# Reporting Summary

Nature Research wishes to improve the reproducibility of the work that we publish. This form provides structure for consistency and transparency in reporting. For further information on Nature Research policies, see our Editorial Policies and the Editorial Policy Checklist.

## Statistics

For all statistical analyses, confirm that the following items are present in the figure legend, table legend, main text, or Methods section.

| n/a | Confirmed | |
|---|---|---|
| ☐ | ☒ | The exact sample size (*n*) for each experimental group/condition, given as a discrete number and unit of measurement |
| ☐ | ☒ | A statement on whether measurements were taken from distinct samples or whether the same sample was measured repeatedly |
| ☐ | ☒ | The statistical test(s) used AND whether they are one- or two-sided<br>*Only common tests should be described solely by name; describe more complex techniques in the Methods section.* |
| ☒ | ☐ | A description of all covariates tested |
| ☒ | ☐ | A description of any assumptions or corrections, such as tests of normality and adjustment for multiple comparisons |
| ☐ | ☒ | A full description of the statistical parameters including central tendency (e.g. means) or other basic estimates (e.g. regression coefficient) AND variation (e.g. standard deviation) or associated estimates of uncertainty (e.g. confidence intervals) |
| ☐ | ☒ | For null hypothesis testing, the test statistic (e.g. *F*, *t*, *r*) with confidence intervals, effect sizes, degrees of freedom and *P* value noted<br>*Give P values as exact values whenever suitable.* |
| ☒ | ☐ | For Bayesian analysis, information on the choice of priors and Markov chain Monte Carlo settings |
| ☒ | ☐ | For hierarchical and complex designs, identification of the appropriate level for tests and full reporting of outcomes |
| ☒ | ☐ | Estimates of effect sizes (e.g. Cohen's *d*, Pearson's *r*), indicating how they were calculated |

*Our web collection on statistics for biologists contains articles on many of the points above.*

## Software and code

Policy information about availability of computer code

| | |
|---|---|
| Data collection | All the sequencing data of COOLAIR and 18S rRNA were run on PacBio Sequel 3.0; The sequencing data of TenA, cspA and RRE61 were run on Illumina NextSeq 500; All QPCR reactions were run with LightCycler® 480; No software was used for other data; |
| Data analysis | For raw reads processing, ccs (https://github.com/PacificBiosciences/ccs) and Lima 1.11.0.<br>For sequence alignment, BLASR v5.3.3.<br>For RNA secondary structure analysis, ViennaRNA2.5.1, CONTRAFold 2.02 (http://contra.stanford.edu/contrafold/download.html) and CentroidFold 0.0.16 (https://github.com/satoken/centroid-rna-package).<br>For visualization of RNA secondary structure, VARNA v.3.93 and RNArtist (https://github.com/fjossinet/RNArtist).<br>For generating SHAPE mutation profile plots, R version 4.0.2.<br>For local base-pair probability analysis, R package astrochron v1.0.<br>For conformation analysis, Determination of the Variation of RNA structure conformation (DaVinci)(https://github.com/DingLab-RNAstructure/smStructure-seq) and Forgi 2.1.2.<br>For figure construction, Adobe Illustrator CC 2021.<br>For quantitative Real-Time PCR data analysis, Microsoft Excel for Mac v16.50. |

For manuscripts utilizing custom algorithms or software that are central to the research but not yet described in published literature, software must be made available to editors and reviewers. We strongly encourage code deposition in a community repository (e.g. GitHub). See the Nature Research guidelines for submitting code & software for further information.

## Data

Policy information about availability of data

All manuscripts must include a data availability statement. This statement should provide the following information, where applicable:

- Accession codes, unique identifiers, or web links for publicly available datasets
- A list of figures that have associated raw data
- A description of any restrictions on data availability

Sequencing data has been deposited in the Sequence Read Archive (SRA) (https://dataview.ncbi.nlm.nih.gov/object/PRJNA749291?reviewer=ql7br4e2j6n6vgovvp0cg3r5lh) under BioProject ID number PRJNA749291. A full list of DNA oligos, PCR primers and COOLAIR reference sequences is available in Supplementary Table 1. The raw data of RNA expression level, RT-PCR and ChIRP-qPCR that support the findings of this study are available in Source Data. Uncropped Images of EMSA and RT-PCR are available in Supplementary Figure 1. The TAIR (The Arabidopsis Information Resource) accession numbers for the genes analysed in this study are FLC (At5g10140) and COOLAIR (At5g01675). Standard reference genes EF1alpha (At5g60390), PP2A (At1g13320) and UBC (At5g25760) for gene expression were used for normalization.

# Field-specific reporting

Please select the one below that is the best fit for your research. If you are not sure, read the appropriate sections before making your selection.

☒ Life sciences ☐ Behavioural & social sciences ☐ Ecological, evolutionary & environmental sciences

For a reference copy of the document with all sections, see nature.com/documents/nr-reporting-summary-flat.pdf

# Life sciences study design

All studies must disclose on these points even when the disclosure is negative.

| | |
|---|---|
| Sample size | No statistical approach was used to predetermine sample size. Sample sizes were determined based on previous publications on similar previous experiments. The determined sample size was adequate as the differences between experimental groups was significant and reproducible.<br>RNA structure probing experiments (Yang, M. et al. Intact RNA structurome reveals mRNA structure-mediated regulation of miRNA cleavage in vivo. Nucleic Acids Res. 48, 8767–8781 (2020)).<br>RT-qPCR (Zhu, P., Lister, C. & Dean, C. Cold-induced Arabidopsis FRIGIDA nuclear condensates for FLC repression. Nature 599, 657–661 (2021).)<br>ChIRP-qPCR (Csorba, T., Questa, J. I., Sun, Q. & Dean, C. Antisense COOLAIR mediates the coordinated switching of chromatin states at FLC during vernalization. Proc. Natl. Acad. Sci. U. S. A. 111, 16160–16165 (2014).)<br>EMSA (Maldonado, R., Filarsky, M., Grummt, I. & Längst, G. Purine- and pyrimidine-triple-helix-forming oligonucleotides recognize qualitatively different target sites at the ribosomal DNA locus. RNA 24, 371–380 (2018).) |
| Data exclusions | No data was excluded from analysis. |
| Replication | The RNA structure experiments were performed with two independent biological replicates. The reproducibility of smStructure-seq has been confirmed by the positive control 18S rRNA (Pearson correlations of 0.95 with P value < 2.2e-16) that has been mentioned in Main Text. The reproducibility of RNA structural experiments has also been validated in the Rebuttal Fig. 4 (Supplementary Information of Peer Review Files). All key experimental findings in the structural mutation analysis were reproduced in more than three independent biological repeats with multiple technical replicates. Main conclusions were confirmed in different assays, including structural analysis in natural accession line, expression level analysis, ChIRP assay and genetic assays in transgenic COOLAIR structural mutants. |
| Randomization | Plants of different genotypes were grown side by side to minimize unexpected environmental variations during growth and experimentation. RNA structure probing was carried out in parallel on the same ThermoMixer (Eppendorf), with minimum covarying factors. Seedlings at the same developmental stage were collected and assessed randomly for each genotype/treatment. For ChIRP-qPCR, multiple seedlings were randomly collected from different plates for each replicate. For RNA expression, multiple, randomly selected individual plants were collected from a plate for each replicate. |
| Blinding | Blinding was not necessary for the molecular biology techniques, where bias could not be introduced as samples were treated together and identically. For bioimaging, the same settings were used for all comparisons. |

# Reporting for specific materials, systems and methods

We require information from authors about some types of materials, experimental systems and methods used in many studies. Here, indicate whether each material, system or method listed is relevant to your study. If you are not sure if a list item applies to your research, read the appropriate section before selecting a response.

## Materials & experimental systems

| n/a | Involved in the study |
| --- | --- |
| ☒ | ☐ Antibodies |
| ☒ | ☐ Eukaryotic cell lines |
| ☒ | ☐ Palaeontology and archaeology |
| ☒ | ☐ Animals and other organisms |
| ☒ | ☐ Human research participants |
| ☒ | ☐ Clinical data |
| ☒ | ☐ Dual use research of concern |

## Methods

| n/a | Involved in the study |
| --- | --- |
| ☒ | ☐ ChIP-seq |
| ☒ | ☐ Flow cytometry |
| ☒ | ☐ MRI-based neuroimaging |
